# Donor Bladder Inclusion in Kidney Transplantation: A Review of the Literature and our Clinical Experience

**DOI:** 10.1007/s11934-026-01328-1

**Published:** 2026-04-01

**Authors:** Gaetano Ciancio, Jeffrey J. Gaynor, Javier Gonzalez, Henry Ocando Nava, Matthew Gaynor, Armando Salim Munoz Abraham, Yuri Kim, Junichiro Sageshima, Mahmoud Morsi

**Affiliations:** 1 Department of Surgery, University of Miami Miller School of Medicine, Jackson Memorial Hospital, 1801 NW 9th Ave, 7th Floor, Miami, FL 33136 USA; 2 Department of Urology, University of Miami Miller School of Medicine, Jackson Memorial Hospital, Miami, FL USA; 3 Miami Transplant Institute, University of Miami Miller School of Medicine, Jackson Memorial Hospital, Miami, FL USA; 4 Servicio de Urología, Unidad de Transplante Renal, Hospital General Universitario Gregorio Marañón, Madrid, Spain; 5 Department of Surgery, Division of Transplant Surgery, University of California Davis School of Medicine, Sacramento, CA USA

**Keywords:** Kidney transplantation, Donor bladder inclusion, Bladder patch, Entire bladder

## Abstract

**Purpose of the Review:**

This review examines bladder inclusion as a reconstructive option in kidney transplantation, particularly when incorporated as part of a pediatric en bloc kidney transplant. With careful patient selection, partial or complete bladder transplant can be performed safely. The technique is relatively straightforward and provides a functionally reliable method of urinary reconstruction and donor ureteral implantation.

**Recent Findings:**

For decades, bladder transplantation was considered technically challenging because of the complex pelvic vascular anatomy, the difficulty of maintaining adequate blood supply, and challenges related to nerve regeneration and function voiding. Despite these obstacles, several attempts of bladder transplantation have been reported in the literature with varying functional outcomes. Notably, one case described transplantation of the entire bladder with sustained function up to 17 years after transplantation.

**Summary:**

Donor bladder inclusion in pediatric en bloc kidney transplantation offers a reliable and technically straightforward approach to ureteral and urinary tract reconstruction in complex kidney transplant recipients. The technique is reproducible, avoids vascular reconstruction, and may have expanded indications in patients requiring at the same time a neobladder or bladder augmentation.

**Supplementary Information:**

The online version contains supplementary material available at 10.1007/s11934-026-01328-1.

## Introduction

Transplant surgery has progressed at a remarkable rate in recent decades. Solid-organ transplantation, once deemed nearly impossible for many intra-abdominal organs, is now routinely performed in complex procedures such as multivisceral transplantation [1]. The uterus, which was once considered extraordinarily difficult to transplant, is now within the realm of standard practice [2]. At present, virtually all intra-abdominal organs have been shown to be transplantable with the notable exception of the urinary bladder.

Bladder transplantation has historically been regarded as largely experimental, and concerns regarding technical complexity, vascular supply, and functional recovery led many to believe that it would be impossible to be successfully performed in clinical transplantation [3].

Atala et al. conducted the first clinical trial in non-transplant patients involving the creation of a bladder using autologous tissue. In this study, patients with bladder dysfunction due to myelomeningocele underwent bladder augmentation using cultured urothelial and smooth muscle cells derived from their own tissue. These cells were seeded onto a biodegradable bladder-shaped scaffold composed of a collagen and polyglycolic acid composite. This clinical trial represented a major milestone and marked the beginning of clinical bladder tissue engineering [4, 5]. However, subsequent outcomes were not sufficiently consistent or durable to support widespread clinical implementation [6].

Gutierres Calzada et al. [7] is one of the very early clinical reports describing transplantation of kidneys together with the donor bladder as a single en bloc graft. It is historically interesting, because it explored the use of an extremely small, anencephalic infant donor for an adult recipient. Unfortunately, the transplant did not achieve durable success, and the technique was abandoned. Although their result was unsuccessful, the paper was important, because it demonstrated several ideas that influenced later transplantations.

Specifically, pediatric en bloc kidney transplantation with bladder inclusion was first performed at our transplant institute in 2006 in a pediatric recipient followed by an adult recipient [8, 9].

In December 2006, we performed (and subsequently reported) the first successful transplant of approximately 90% of the donor urinary bladder (almost the entire donor bladder transected at the level of the bladder neck of the donor) into an adult kidney transplant recipient. The recipient had a severely contracted bladder secondary to long-standing end-stage kidney disease (ESKD) and had been maintained on dialysis for 10 years prior to transplant. The donor bladder was procured along with the kidneys en bloc from a 13-month-old pediatric donor, including the trigone and both ureters. During the transplant, the graft bladder was anastomosed to the remnant of the recipient’s native bladder, providing augmentation to restore adequate capacity and compliance [9].

The post-operative course was uncomplicated, with progressive improvement in urinary capacity and function. The patient achieved normal voiding patterns and maintained normal kidney graft function through 13 years of post-transplant follow-up (this patient was no longer being followed at our center beyond that time). Serial imaging studies showed normal bladder capacity and compliance, with no evidence of leakage, infection, or rejection [9]. This adult case demonstrated the technical feasibility and long-term functional success of the donor bladder being transplanted in conjunction with pediatric en bloc kidney transplantation. It also added clinical evidence supporting the potential role of donor bladder tissue for functional urinary tract reconstruction in patients with ESKD having a small, contracted bladder [9].

Although successful bladder transplantation has previously been reported, long-term outcomes remain insufficiently documented. More recently, the first full human bladder transplant (in combination with a kidney) at UCLA Health was performed in May 2025 [10].

The current review presents long-term outcomes of donor bladder inclusion in the setting of pediatric en bloc kidney transplantation, with the longest follow-up reaching 17 years post-transplant in one case [11].

## Lessons Learned from our Experience

We conducted a review of our consecutive series of 15 pediatric en bloc kidney transplant cases that incorporated the donor bladder, performed either as an isolated kidney transplant or in combination with pancreas, liver, or multivisceral transplantation [8, 9, 12–14]. The complete donor bladder was utilized for urinary tract reconstruction in 5 recipients with a small and/or contracted native bladder (3 due to congenital reasons; 2 due to long-term dialysis). The vascular supply of the donor bladder is derived from the ureter, thereby obviating the need for separate vascular anastomosis or reconstruction. None of the recipients developed donor bladder rejection or experienced any urological complications during the post-transplant follow-up. While the use of ureteral stents was determined at the discretion of the transplant surgeon, 7 recipients who received initial stent placement were transplanted prior to 2014, and 8 recipients who did not receive initial stent placement were transplanted since 2016. The surgical technique described here is technically straightforward and can readily be reproduced at other centers [15–17]. Furthermore, the approach may have broader applicability in patients with ESKD who require bladder augmentation or neobladder reconstruction. In selected extreme cases, the donor bladder neck could be anastomosed directly to the skin as urinary stoma. Our cohort includes the longest reported follow-up to date, 17 years in a pediatric recipient of this procedure [8, 11].

## Methods

This study was approved by the University of Miami Institutional Review Board and adheres to the ethical principles of the Helsinki Declaration, as revised in 2013.

### Donor Procurement

**Fig. 1:**
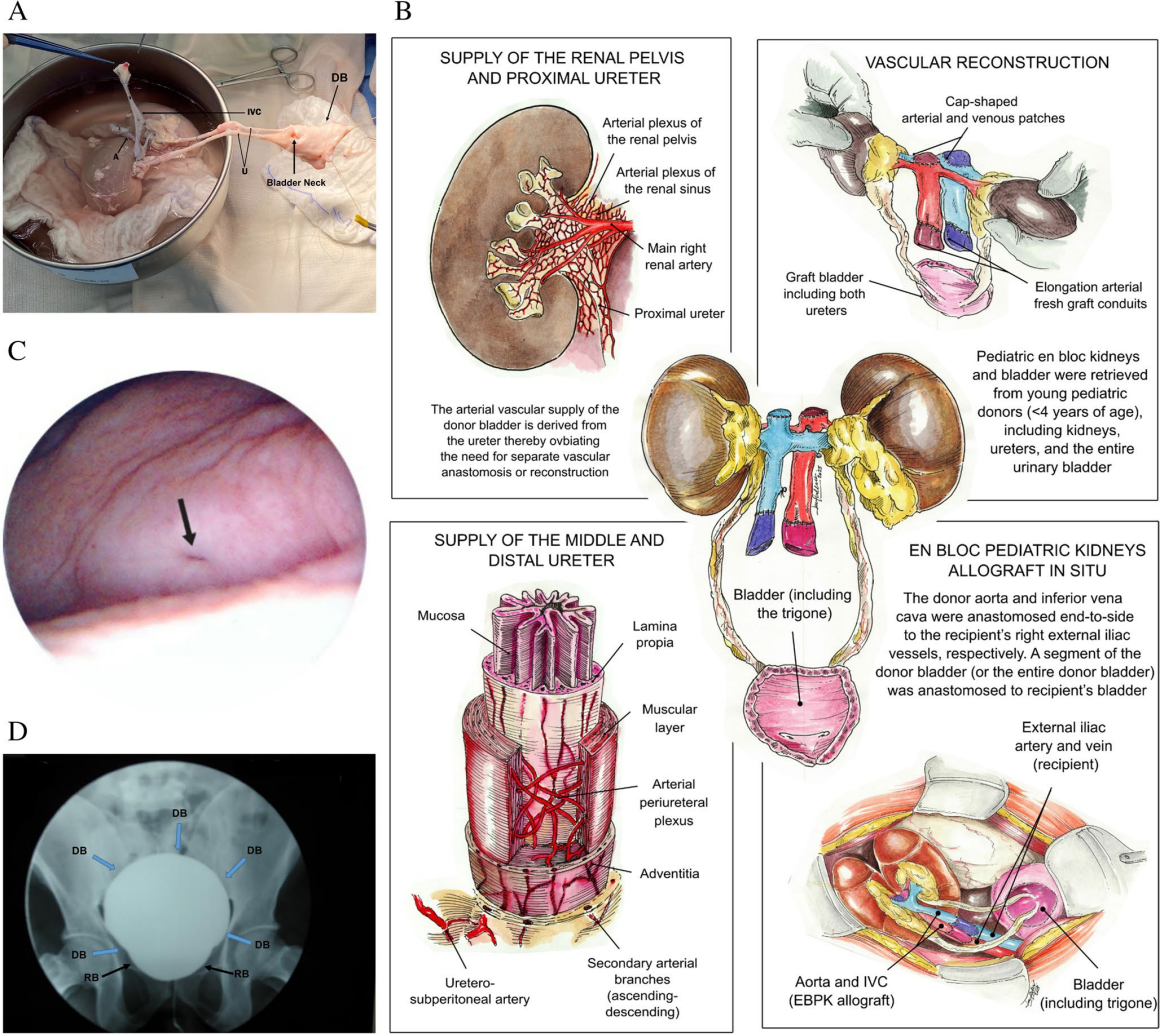
**A** Pediatric en bloc kidneys with the entire donor bladder (DB) and bladder neck (8-month-old infant), both ureters (U), Inferior Vena Cava (IVC), Aorta (A) are shown. The bladder is rotated, because the pediatric en bloc kidneys are also rotated, positioning the IVC on the left and the A on the right to align with the recipient’s right external iliac vessels, specifically, the external iliac artery (lateral) and the vein (medial). **B** Top left: Showing the arterial blood supply of the renal pelvis and proximal ureter, and Left below: middle and distal ureter arterial blood supply. Top Right: Showing the vascular reconstruction of the donor aorta and inferior vena cava (IVC) with donor vessels including a cap shaped vascular patches on top of the aorta and IVC, Right below: en bloc pediatric kidneys transplant including the bladder with both ureter. Middle: pediatric en bloc kidney ready for the transplant with the entire bladder. **C** Cystoscopy of transplant bladder, transplant ureteral orifice (black arrow). **D** Cystogram showing the recipient bladder (RB) and donor bladder (DB) at 18 months after transplantation

This is the most vital part of the procedure. Pediatric en bloc kidneys and bladder were retrieved from young pediatric donors (< 4 years of age), which included kidneys, ureters, and the entire urinary bladder (Fig. 1A). The donor aorta and inferior vena cava were preserved for arterial and venous anastomoses. The entire bladder was obtained with its distal ureters intact, and the bladder neck was also included and preserved in cold storage solution. Of note, it is important to have extra vessels available for performing vascular reconstruction [12]. The aorta and inferior vena cava (IVC) were extended using additional donor vessels, and a cap-shaped vascular patch was placed over the proximal ends of the aorta and IVC to prevent stricture of the renal arteries and veins (Fig. 1B) [12, 18].

Following allograft recovery (including en bloc kidneys retrieved for performing a simultaneous pancreas-kidney transplant), all donors kidneys were connected to a hypothermic renal preservation machine. Kidney allografts were arterially (Aorta) cannulated and perfused with Kidney Perfusion Solution. Perfusion temperature was set at 4 °C, and systolic perfusion pressure was initially fixed at 30 mmHg, with adjustment made after 30 min to obtain a flow goal according to kidney size. Pumping flow (ml/min) and resistance indices (mmHg/ml/min) were serially monitored. All kidneys received Mannitol 12.5 g/50 ml/hr until time of transplant [9, 12, 13, 19].

In the other retrieval cases, i.e., simultaneous liver-kidney and combined multivisceral-kidney, preservation with cold storage but without machine perfusion was used.

### The Surgical Procedure

A standard right lower quadrant Gibson incision was made to access the retroperitoneal space. The peritoneum was mobilized medially exposing the right iliac vessels. Blunt dissection was performed posteriorly toward the right renal fossa to complete exposure. A Bookwalter retractor was positioned to maintain optimal visualization throughout the procedure. The donor aorta and inferior vena cava were anastomosed end-to-side to the recipient’s right external iliac artery and vein using 6 − 0 and 5 − 0 polypropylene sutures, respectively. A segment of the donor bladder (or the entire donor bladder but transected at the level of the bladder neck) that included the trigone and both ureters was anastomosed to recipient’s bladder using running 4 − 0 polydioxanone (PDS), thereby simplifying urinary drainage and preserving natural anti-reflux mechanisms. This specific technique was used in kidney-bladder and simultaneous pancreas-kidney-bladder recipients. In pediatric recipients of either pediatric en bloc liver-kidneys with bladder inclusion, or pediatric en bloc multivisceral-kidneys with bladder inclusion, the anastomosis of the donor bladder was performed in two layers of running 5 − 0 PDS suture [8, 9, 12–14].

Additionally, the decision to place ureteral stents at the time of transplant was left to the discretion of the transplant surgeon.

It should be noted that in cases where the recipient bladder functioned normally, only a segment of the donor bladder (approximately 40%-50% of the donor bladder) was utilized in the transplant. Conversely, in cases where the recipient had a small, contracted bladder and required bladder augmentation (in our cases due either to congenital reasons or as a consequence of long-term dialysis), the entire donor bladder but transected at the level of the bladder neck (thus, approximately 90% of the donor bladder) was utilized in the transplant.

It should also be noted that in both cases, whether a donor bladder segment or nearly the entire donor bladder is utilized and transplanted, the donor bladder’s vascular supply is initially derived directly from the donor kidneys and ureters. Specifically, during organ procurement, all of the native segmental branches from the aorta, renal, gonadal, and iliac arteries are sacrificed. Thus, in the transplanted kidney, nearly all arterial and venous perfusion of the ureter originate from the renal pelvis and periureteral branches of the renal artery, extending distally toward the bladder. As the vascular supply of the donor bladder is initially derived from the donor kidneys and ureters, this obviates the need for performing a separate vascular anastomosis or reconstruction of the donor bladder.

### Postoperative Management and Follow-up

All recipients received induction therapy and standard maintenance immunosuppressive therapy consisting of a calcineurin inhibitor, mycophenolate acid, and corticosteroids. Bladder function was monitored with ultrasound, cystogram and serum creatinine level. Postoperatively, the Foley catheter was maintained for 14 days in pediatric and adult recipients of pediatric en bloc kidneys with donor bladder, as well as in recipients undergoing transplantation of simultaneous pediatric pancreas and en bloc kidneys with donor bladder. In contrast, the Foley catheter was maintained for 21 days in pediatric recipients undergoing transplantation of either pediatric en bloc liver-kidneys and donor bladder or multivisceral-kidneys and donor bladder. Patient follow-up ranged from 6 months to 17 years post-transplant.

## Our Experience with Donor Bladder Inclusion

### In Pediatric En Bloc Kidney Transplantation (*N* = 6)

Selected baseline characteristics and kidney graft outcomes for each patient appear in Table 1. The first successful transplant of pediatric en bloc kidneys with donor bladder inclusion was performed in early 2006 in a 12-month-old female recipient with a congenitally very small bladder; donor age was 2 years (see Table 1). Due to poor bladder capacity of the recipient, approximately 90% of the entire donor bladder was transplanted en bloc with the 2 donor kidneys and ureters [8]. Long-term functional outcomes were recently published [11], and the 17-year follow-up of this patient included an evaluation of entire-bladder function at 17 1/2 years of age. The patient has remained free of urinary tract infections throughout the follow-up period. Cystoscopy demonstrated a viable transplanted bladder with a well-perfused mucosa. Over time, the native bladder expanded, now forming more than half of the total bladder wall. Urodynamic assessment revealed preserved bladder compliance (43 mL/cm H₂O) and maintained native bladder contractility. However, prolonged voiding time and post-void residual urine were observed, findings consistent with detrusor underactivity. No vesicoureteral reflux across the donor ureterovesical junctions was identified. The recipient was advised to continue timed and double voiding to ensure complete bladder emptying. In conclusion, pediatric en bloc kidney transplantation incorporating a bladder patch that includes the entire trigone represents a feasible and durable surgical option for kidney transplant recipients with severely reduced bladder capacity, offering sustained function and low complication rates during long-term follow-up [11].

**Table 1:** Selected baseline characteristics and kidney graft outcomes for each patient

DOT^1^	Pediatric En Bloc Kidneys Transplanted	Age (y)	Sex	Donor Age (y)	Donor Wt (kg)	% of Bladder Included (Reason for Augmentation)	Kidney Graft Outcome (y)
4/1/06	Alone	12 m	F	2	14.3	90% (congenital)	FuncGr at 17y
12/9/06	Alone	45	M	13 m	9	90% (long-term dialysis)	FuncGr at 13y
8/15/09	Alone	28	F	2	12	90% (long-term dialysis)	DWFG at 14 m
3/2/17	Alone	47	F	10 m	8	40–50%	GrFail at 6.5y
3/14/20	Alone	33	M	8 m	11.8	40–50%	FuncGr at 5.5y
3/9/25	Alone	45	M	3	15	40–50%	FuncGr at 6 m
3/19/09	SPK	32	F	14 m	10	40–50%	GrFail at 4.2y
3/20/10	SPK	35	F	18 m	10	40–50%	DWFG at 4.2y
3/15/13	L-K	3y 9 m	M	4 m	6.4	40–50%	FuncGr at 10y
12/6/19	L-K	21 m	F	22 m	11.1	90% (congenital)	FuncGr at 6y
8/1/13	MVT	7	F	3	17.7	40–50%	FuncGr at 12y
2/23/16	MVT	12 m	M	6 m	7.3	90% (congenital)	FuncGr at 9y
7/9/16	MVT	3y 7 m	M	12 m	12.2	40–50%	DWFG at 2 m
11/7/20	MVT	5	M	20 m	15.8	40–50%	FuncGr at 4.3y
10/21/24	MVT	8	M	3	14.5	40–50%	DWFG at 2d

Abbreviations: *DOT*, date of transplant; *DWFG*, death with a functioning graft; *L-K*, liver-kidney; *MVT*, multivisceral transplant including en bloc kidneys and bladder; *SPK*, simultaneous pancreas-kidney

^1^Of note, stents were placed at transplant in all patients transplanted prior to 2014; none of the patients transplanted since 2016 received stent placement

We also reported the first successful transplant of pediatric en bloc kidneys with donor bladder inclusion in an adult (45-year-old) male recipient; donor age was 13 months [9]. This recipient had been on long-term hemodialysis and presented with a small, contracted bladder secondary to long-standing anuria. The donor bladder was included here to avoid performing bilateral ureteroneocystotomies of extremely small pediatric ureters as well as to augment the recipient’s native bladder, with approximately 90% of the entire donor bladder being transplanted en bloc with the 2 donor kidneys and ureters. Cystoscopy evaluations at 3mo and 18mo post-transplant demonstrated a viable and well-vascularized bladder segment with normal donor mucosa and no evidence of ischemia (Fig. 1C). Cystography revealed no vesicoureteral reflux (Fig. 1D). The transplanted bladder segment initially received perfusion through the donor ureteral vasculature, later developing neovascularization from the recipient (native) bladder. This technique not only eliminates the need for performing complex bilateral ureteroneocystostomies but also allows for augmentation of bladder capacity, providing a promising surgical alternative in selected patients with a very small, contracted bladder or in need of bladder augmentation [9]. The third patient who received pediatric en bloc kidneys with donor bladder inclusion was a 28-year-old female recipient with ESKD due to systemic lupus erythematosus (SLE) who had been on hemodialysis for approximately 6 years; donor age was 2 years. Pre-operatively, the recipient was noted to have a small, contracted bladder; thus, approximately 90% of the donor bladder was included to achieve urinary tract reconstruction. At 12mo post-transplant, cystoscopy and biopsy of the donor bladder demonstrated no histological evidence of rejection - only some mild mucosal edema was observed. Unfortunately, the patient died with a functioning graft at 14mo post-transplant due to sepsis with a 12mo serum creatinine of 0.87 mg/dL.

The fourth recipient was a 47-year-old female with ESKD due to focal segmental glomerulosclerosis (FSGS) who had been maintained on hemodialysis for approximately 6 years; donor age was 10 months. Since in this case the recipient had reasonable bladder compliance, only 40%-50% of the donor bladder was included. At 12mo post-transplant, the patient’s serum creatinine remained stable at 0.8 mg/dL. Unfortunately, she later developed antibody-mediated rejection that was refractory to antirejection therapy, ultimately resulting in graft loss and return to dialysis. She is currently awaiting a second kidney transplant.

The fifth recipient was a 33-year-old male with ESKD secondary to biopsy-proven glomerulonephritis and on hemodialysis for approximately 3 years; donor age was 8 months. Since this recipient had reasonable bladder capacity, only 40%-50% of the donor bladder was included. At 5 years of post-transplant follow-up, serum creatinine was 0.62 mg/dL, and there were no voiding issues [12].

We recently performed our sixth pediatric en bloc kidney transplant incorporating a donor bladder segment. The recipient was a 45-year-old male with ESKD due to biopsy-proven FSGS who had been on peritoneal dialysis for approximately 1 ½ years; donor age was 3 years. Since this recipient had reasonable bladder capacity, only 40%-50% of the donor bladder was included. At 6mo post-transplant, the recipient’s serum creatinine was 1.1 mg/dL, with no evidence of urinary voiding complications.

### In Simultaneous Pancreas and Pediatric En Bloc Kidney Transplantation (*N* = 2)

Sageshima et al. [13] reported two cases of adult female recipients (ages 32 and 35 years) undergoing simultaneous pancreas and pediatric en bloc kidney transplantation from very small donors (14- and 18-months-old) using a donor bladder segment in performing the ureteral/vesical reconstruction. Our technique involved procurement of the donor kidneys en bloc with both ureters and a segment of donor bladder (the bladder patch) such that no ureteroneocystostomies for very small donor ureters were required. Since both recipients had reasonable bladder capacity, only 40%-50% of the donor bladder was included in these 2 cases.

One of the takeaways of this report is the current underutilization of very small pediatric donors for combined pancreas-kidney transplantation because of concerns about insufficient islet cell mass, donor size, and what was previously thought as being technically complicated-to-perform urinary tract reconstruction. The use of a segment of donor bladder technique offers a surgical solution to the challenge of implanting very small ureters: using the donor bladder segment (with ureterovesical junctions intact), technical complexity and risk of urinary complications are greatly reduced.

At 2 years post-transplant, the first SPK recipient still had sustained euglycemia without requiring exogenous insulin, showing that even very small pediatric donors can provide sufficient islet mass for adult recipients when appropriately managed. The first SPK recipient then became insulin dependent by 3 year post-transplant, while the second SPK recipient became insulin dependent by 6mo post-transplant. In fact, the second SPK recipient developed pancreas graft failure (due to acute rejection/graft sclerosis) at 11mo post-transplant. Both patients continued dialysis independence through 3 year post-transplant, demonstrating that pediatric en bloc pediatric kidneys can grow and maintain normal renal function for an extended time post-transplant. However, the first SPK recipient developed kidney graft failure at 4.2 year post-transplant due to chronic allograft injury (following numerous acute rejection episodes), and the second SPK recipient died with a functioning kidney graft (likely due to a cardiovascular event) at 4.2 year post-transplant. The absence of any urinary tract complications in these 2 patients also validates the surgical concept of transplanting the donor bladder segment with an intact trigone, preserving the native ureterovesical junctions and effectively avoiding the risks inherent to performing multiple ureteral anastomoses in very small donor ureters [13]. This work demonstrates the feasibility of using very small pediatric donors for combined pancreas and kidney transplantation in adults, thereby expanding the donor pool.

### In Pediatric En Bloc Simultaneous Liver-Kidney Transplantation (*N* = 2)

In the report by Kunzler de Oliveira Maia et al. [14], four pediatric recipients underwent combined organ transplantation using pediatric donor en bloc kidneys with donor bladder inclusion. One of these 4 patients was a 3-year-9-month-old male, with a history of autosomal dominant polycystic kidney disease (ADPKD) and congenital hepatic fibrosis. He began peritoneal dialysis at age 2 months and transitioned to hemodialysis at age 4 months. The recipient underwent pediatric en bloc simultaneous liver and kidney transplantation with donor bladder inclusion from a 4-month-old donor; thus, no ureteroneocystostomies for very small donor ureters were required. This recipient had a reasonably compliant bladder; thus, approximately 40%-50% of the donor bladder was included here. At 10 years post-transplant, this patient remains free of any urinary complications involving the donor bladder as well as being dialysis-free with excellent graft function.

The second recipient of a pediatric en bloc simultaneous liver and kidney transplantation with bladder inclusion was a 21-month-old female, not included in the Kunzler de Oliveira Maia et al. [14] report. Of note, this recipient’s bladder was small and contracted since birth (congenital); thus, approximately 90% of the donor bladder was included as part of performing bladder augmentation. At 6 years post-transplant, this patient remains free of any urinary complications involving the donor bladder as well as being dialysis-free with excellent graft function.

### In Pediatric En Bloc Multivisceral Transplantation (*N* = 5)

Three of the 4 pediatric recipients reported by Kunzler de Oliveira Maia et al. [14] underwent pediatric en bloc multivisceral transplantation that included en bloc kidneys and a segment of donor bladder. In such complex settings, particularly in pediatric recipients, performing separate ureteral anastomoses for very small donor ureters carries a significant risk of developing either ischemia/necrosis (which would include urinary leakage) or ureteral stricture/obstruction (or both). Use of a donor bladder segment or the entire bladder with intact ureterovesical junctions offers a practical surgical solution, reducing technical complexity and minimizing the risk of urological complications. The donor bladder is then anastomosed directly to the recipient’s bladder, simplifying urinary drainage and preserving natural anti-reflux mechanisms.

Notably, the first patient (patient #2 in the Kunzler de Oliveira Maia et al. [14] report) was a 7-years old female; donor age was 3 years. This patient had a reasonably compliant bladder; thus, approximately 40%-50% of the donor bladder was included here. The second patient was a 12-month-old male; donor age was 6 months. This patient underwent a Mitrofanoff catheterizable stoma procedure 48 h after the multivisceral transplant, using a recipient bladder-based flap. Because this recipient congenitally had a hypoplastic bladder, nearly the entire donor bladder (roughly 90%) was used for urinary tract reconstruction. These first 2 patients were alive with functioning grafts at 12 year and 9 year post-transplant, respectively. The third patient was a 3 year and 7 month-old male who received multivisceral organs including kidneys and bladder from a 12-month-old donor. This patient had a reasonably compliant bladder; thus, approximately 40%-50% of the donor bladder was included here. Unfortunately, this patient died of sepsis at 2mo post-transplant with intestinal graft failure (due to severe acute rejection) but with a functioning kidney allograft and a serum creatinine (at 1mo post-transplant) of 0.29 mg/dL.

Two additional male pediatric patients aged 5-years and 8-years underwent a combined pediatric en bloc multivisceral, double kidney transplant with bladder inclusion. Donor ages were 20 months and 3 years, respectively. Since both patients had reasonably compliant bladders, only 40%-50% of the donor bladders were included, respectively. With follow-up beyond 4 years, one recipient demonstrated excellent graft function, having a serum creatinine level of 0.70 mg/dL at 4 years post-transplant. Unfortunately, the second patient developed irreversible coagulopathy and sepsis, leading to death at 2 days post-transplant.

## Other Successful Attempts of Bladder Transplantation

Flechner et al. [15] reported three cases of pediatric en bloc kidney transplantation into adult recipients using the donor bladder trigone to simplify urinary reconstruction. The donors were aged 11, 21 and 23 months (two males and one female). Recipients of the male donor kidneys recovered well, with normal voiding, no vesi-ureteral reflux, and serum creatinine levels of 1.0 and 1.2 mg/dL at 12mo and 14mo post-transplant, respectively. The recipient of the female donor developed a pelvic abscess, necessitating reconstruction of the ureters and segment of the donor bladder. This recipient recovered with normal voiding and a serum creatinine of 1.2 mg/dL at 9mo post-transplant.

Dogan et al. [16] reported another transplant case of pediatric en bloc kidneys with a partial bladder segment from 1.5-year-old donor into a 12-year-old male recipient with ESKD due to vesicoureteral reflux (VUR) of a solitary kidney. The donor kidneys, ureters and a segment of bladder were transplanted en bloc, augmenting the recipient’s bladder and avoiding the risks associated with performing bilateral ureteroneocystostomies of very small ureters. Over 12mo of follow-up, the recipient maintained normal kidney function and experienced no ureteral complications or any evidence of VUR.

A recent report by Kim et al. [17] described a pediatric en bloc kidney transplant including a donor bladder segment. The donor was a 5-month-old infant weighing 7.2 kg, and the recipient was a 48-old male with ESKD secondary to type 2 diabetes and hypertension, who had been on peritoneal dialysis for 5 years. At 29mo post-transplant, the patient’s serum creatinine level was 0.8 mg/dL, with no reported urinary complications.

## Discussion

Gutierrez Calzada et al. [7] previously reported en bloc kidneys and bladder transplantation from a 3-day-old anencephalic donor into an adult recipient. Although the kidney grafts exhibited limited function and were eventually lost due to refractory rejection, their early postoperative findings provided valuable anatomical insights. In our series, cystoscopy and cystogram evaluations at 3mo and 18mo post-transplant demonstrated findings consistent with those described by Gutierrez Calzada et al. [7], showing uniform mucosal integration throughout the donor-recipient bladder complex. Importantly, there was no evidence of vesicoureteral reflux, confirming the functional integrity of the reconstructed bladder unit [9].

In normal retroperitoneal anatomy, the ureters receive blood supply from multiple arteries forming a continuous longitudinal network [20]. The abdominal ureter derives its arterial supply primarily from branches of the abdominal aorta, as well as from the renal, gonadal, and common iliac arteries, which approach the ureter medially. The distal ureter receives blood from the superior and inferior vesical arteries and, in females, from the uterine artery, all branches of the internal iliac artery. These vessels form an anastomotic plexus along the ureter, providing a rich collateral circulation that permits safe mobilization during surgery when careful dissection from surrounding structures is necessary. The venous and lymphatic drainage of the ureter parallels its arterial supply. Lymphatic channels drain into the internal, external, and common iliac nodes, with the left ureter draining toward the left paraaortic lymph nodes, and the right ureter draining toward the right paracaval and interaortocaval nodes.

This vascular pattern represents normal retroperitoneal anatomy of the native kidney and ureter. However, this pattern differs significantly from the vascular supply of the ureter in renal transplantation, where all of the native segmental branches from the aorta, renal, gonadal, and iliac arteries are sacrificed during organ procurement. In the transplanted kidney, nearly all arterial and venous perfusion of the ureter originate from the renal pelvis and periureteral branches of the renal artery, extending distally toward the bladder. This limited and delicate blood supply makes the transplanted ureter particularly vulnerable to ischemic injury. Understanding this unique vascular dependence is essential when including a donor bladder segment or the entire donor bladder in conjunction with pediatric en bloc kidneys, as it preserves the ureterovesical junctions with their native blood supply, thereby minimizing the risk of ischemia and subsequent urological complications [21].

In their recent review, Kuttymuratov et al. [21] concluded that bladder transplantation remains an experimental procedure without current clinical application. However, the authors did not acknowledge the contributions of several transplant institutions which have already incorporated the donor bladder into their clinical transplant practice [8, 9, 12–17]. Furthermore, while the review suggests that immunological mechanisms underlying bladder transplantation remain poorly understood, it is noteworthy that none of the reported clinical cases have described graft rejection of the bladder component. Consistent with these findings, our own experience similarly indicates an absence of bladder rejection in transplant cases [8, 9, 12–14].

In a brief communication Shah [10] recently reported the first successful human bladder transplant performed (using robotic surgery) at UCLA Health in Los Angeles. The procedure involved transplantation of an entire bladder along with the kidney allograft from an adult deceased donor into a 41-year-old male recipient who had lost both organs due to medical complications and had been on dialysis. He had a small bladder after bilateral nephrectomies and near complete resection of his bladder for urachal adenocarcinoma. The surgical approach included vascular anastomosis of the donor bladder vessels to the recipient’s iliac vessels followed by the donor kidney ureter to the donor bladder being transplanted. This case may represent the first reported full human bladder organ transplant, distinct from earlier reports that utilized partial bladder segment [12–14]. However, some previous publications also described inclusion of the entire bladder in combination with pediatric en bloc kidney transplantation [8, 9]. The UCLA group performed a vascularized bladder (https://www.nytimes.com/2025/05/18/health/bladder-transplanthuman.html) whereas in prior cases [8, 9, 12–17], the donor bladder segment and the entire bladder’s vascular supply was derived from the donor kidneys. We now report long-term follow-up exceeding 5 years post-transplant in 8 of our 15 cases.

Vascularized bladder transplantation represents a promising new frontier in reconstructive urology and transplant surgery, offering hope for patients with irreversible bladder loss. All patients described in these reports had ESKD, and several presented with a small, contracted bladder, a combination that made them ideal candidates for this dual-purpose surgical approach. In contrast, patients with bladder loss due to malignancy are unlikely to benefit from bladder transplantation because of the risks associated with taking lifelong immunosuppressive drugs. In such cases, alternative reconstructive options such as orthotopic neobladder or urinary diversion [22] remain more appropriate. Patients who progress to ESKD and are deemed oncologically eligible for transplantation can benefit from a dual-purpose surgical approach utilizing pediatric en bloc kidneys with the entire donor bladder. Pediatric donors remain underutilized, with a discard percentage of 40.3% for pediatric donors weighing less than 10 kg, markedly higher than the 10.5% amount observed in donors weighing 10–21 kg [23]. Consequently, the availability of suitable organs for these patients is high [24], enabling timely transplantation without the necessity of staged procedures to manage their clinical condition. Our findings confirm the feasibility of this dual-purpose approach with encouraging long-term results [8, 9, 12–14].

## Conclusions

These observations suggest that, with careful patient selection and immunosuppressive management, using the donor bladder in pediatric en bloc kidney transplantation can be performed safely, and it is a straightforward technique with a functionally reliable method of urinary and donor ureteral reconstruction. The procedure eliminates the need for vascular reconstruction, demonstrating long-term viability without rejection or urological complications, and may have expanded indications in patients requiring some type of bladder augmentation or neobladder creation.

## Supplementary Information

Below is the link to the electronic supplementary material.

Supplementary Material 1

## Data Availability

De-identified data will be provided upon reasonable request.

## References

[1] Abu-Elmagd KM, Costa G, Bond GJ, Soltys K, Sindhi R, Wu T, Koritsky DA, Schuster B, Martin L, Cruz RJ, Murase N, Zeevi A, Irish W, Ayyash MO, Matarese L, Humar A, Mazariegos G. Five hundred intestinal and multivisceral transplantations at a single center: major advances with new challenges. Ann Surg. 2009;250(4):567–81. 19730240 10.1097/SLA.0b013e3181b67725

[2] Walter JR, Jungheim ES. Uterus Transplant-The Frontier of Innovative Fertility Treatment. JAMA. 2024;332(10):792–3. 39145970 10.1001/jama.2024.13548

[3] Czarnogórski MC, Koper K, Petrasz P, Vetterlein MW, Pokrywczyńska M, Juszczak K, Drewa T, Adamowicz J. Urinary bladder transplantation in humans—Current status and future perspectives. Nat Rev Urol. 2024;22:175–86. 39304780 10.1038/s41585-024-00935-2

[4] Atala A, Bauer SB, Soker S, Yoo JJ, Retik AB. Tissue-engineered autologous bladders for patients needing cystoplasty. Lancet. 2006;367(9518):1241–6. 16631879 10.1016/S0140-6736(06)68438-9

[5] Atala A. Tissue engineering of human bladder. Br Med Bull. 2011;97:81–104. 21324973 10.1093/bmb/ldr003

[6] Joseph DB, Borer JG, De Filippo RE, Hodges SJ, McLorie GA. Autologous cell seeded biodegradable scaffold for augmentation cystoplasty: phase II study in children and adolescents with spina bifida. J Urol. 2014;191:1389–95. 24184366 10.1016/j.juro.2013.10.103

[7] Gutiérrez Calzada JL, Martínez JL, Baena V, et al. En bloc kidney and bladder transplantation from an anencephalic donor into an adult recipient. J Urol. 1987;138:125. 3298688 10.1016/s0022-5347(17)43017-5

[8] Kato T, Selvaggi G, Burke G, Ciancio G, Zilleruelo G, Hattori M, Gosalbez R, Tzakis A. Partial bladder transplantation with en bloc kidney transplant–the first case report of a ‘bladder patch technique’ in a human. Am J Transpl. 2008;8(5):1060–3. 10.1111/j.1600-6143.2008.02180.x 18312611

[9] Ciancio G, Kato T, Chen L, Sageshima J, Livingstone AS, Burke GW. Transplantation of en bloc pediatric kidneys with a partial bladder segment in an adult recipient. Transpl Int. 2009;22(3):350–3. 19220872 10.1111/j.1432-2277.2008.00815.x

[10] Shah AM. First Human Bladder Transplant. Artif Organs. 2025;49(8):1227–8. 40503649 10.1111/aor.15041

[11] Shirai Y, Miura K, Suzuki M, Moriyama I, Yoshino M, Takagi T, Kato T, Hattori M. Partial bladder transplantation with en bloc kidney transplant-long-term, 17 years, the outcome of a bladder patch technique. Am J Transpl. 2024;24(11):2121–4. 10.1016/j.ajt.2024.07.007 39002782

[12] Gonzalez J, Tekin A, Vincenzi P, Alvarez A, Ciancio G. Transplantation of En Bloc Pediatric Kidneys With a Bladder Segment Patch After a Complex Vascular Reconstruction: A Case Report. Transpl Proc. 2021;53(8):2524–8. 10.1016/j.transproceed.2021.06.009 34247862

[13] Sageshima J, Ciancio G, Chen L, Selvaggi G, Nishida S, Akpinar E, Nesher E, Romano A, Misawa R, Burke GW 3. Combined pancreas and en bloc kidney transplantation using a bladder patch technique from very small pediatric donors. Am J Transpl. 2010;10(9):2168–72. 10.1111/j.1600-6143.2010.03229.x 20883550

[14] Kunzler de Oliveira Maia F, Tekin A, Nicolau-Raducu R, Beduschi T, Selvaggi G, Vianna R, Ammar Al Nuss M, González J, Gaynor JJ, Ciancio G. Use of pediatric donor en bloc kidneys along with bladder segment in pediatric liver-kidney and multivisceral-kidney transplantation. Pediatr Transpl. 2020;24(1):e13596. 10.1111/petr.13596 31605438

[15] Flechner SM, Saad IR, Tiong HY, Rabets J, Krishnamurthi V. Use of the donor bladder trigone to facilitate pediatric en bloc kidney transplantation. Pediatr Transpl. 2011;15(1):53–7. 10.1111/j.1399-3046.2010.01410.x 20946194

[16] Dogan M, Tugmen C, Kebapci E, Yildirim U, Karaca C, Alparslan C, Yavascan O, Aksu N. En-bloc pediatric kidney transplantation together with a partial bladder segment: a case report. Pediatr Nephrol. 2011;26(5):805–7. 21212986 10.1007/s00467-010-1743-3

[17] Kim SH, Yu HC, Hwang HP, Lee S. En-bloc Pediatric kidney transplant to Adult Recipient with two Different Urterovesical Anastomosis Techniques. Am J Case Rep. 2019;20:517–21. 30982058 10.12659/AJCR.914290 PMC6476232

[18] Garcia LE, Gonzalez J, Serena G, Al-Nuss MA, Morsi M, DeFaria W. Ciancio G.Donor vascular patch for reconstruction of the aorta and inferior vena cava in pediatric en bloc kidney transplantation. Actas Urol Esp (Engl Ed). 2019;43(9):515–6. 31235248 10.1016/j.acuro.2019.05.003

[19] Ciancio G, Gaynor JJ, Sageshima J, Chen L, Roth D, Kupin W, Guerra G, Tueros L, Zarak A, Hanson L, Ganz S, Ruiz P, O’Neill WW, Livingstone AS. Burke GW 3rd. Favorable outcomes with machine perfusion and longer pump times in kidney transplantation: a single-center, observational study. Transplantation. 2010;90(8):882–90. 20703178 10.1097/TP.0b013e3181f2c962

[20] Leslie SW, Sajjad H. StatPearls [Internet]. StatPearls Publishing; Treasure island (FL): Aug 8, 2023. Anatomy, abdomen and pelvis, renal artery. Publishing Jan 2026. Available from: https://www.ncbi.nlm.nih.gov/books/NBK459158/29083626

[21] Kuttymuratov G, Saliev T, Ainakulov A, Ayaganov A, Oshakbayev K, Zharassov D, Tuleuzhan A, Uderbayev N. Kidney and Bladder Transplantation: Advances, Barriers, and Emerging Solutions. Med (Kaunas). 2025;61(6):1045. 10.3390/medicina61061045 PMC12195452 40572733

[22] Abusal F, Alawadi A, Akpala A, Obeidat S, Al-Omari N, Alsharief M, Shalaby A, Shaukat Z, Sarkar D. Long-Term Complications and Quality of Life After Urinary Diversion for Bladder Cancer: A Systematic Review and Meta-Analysis. Cureus. 2025;17(5):e84744. 40551917 10.7759/cureus.84744 PMC12184956

[23] Pelletier SJ, Guidinger MK, Merion RM, Englesbe MJ, Wolfe RA, Magee JC, Sollinger HW. Recovery and utilization of deceased donor kidneys from small pediatric donors. Am J Transpl. 2006;6(7):1646–52. 10.1111/j.1600-6143.2006.01353.x 16827866

[24] Troppmann C, Santhanakrishnan C, Fananapazir G, Sageshima J, Troppmann KM, Perez RV. Short- and long-term outcomes of Kidneys Transplant From Very Small (≤ 15 kg) Pediatric Donors With Acute Kidney Injury. Transplantation. 2021;105(2):430–5. 32217942 10.1097/TP.0000000000003230

